# Matching the Best Viewing Angle in Depth Cameras for Biomass Estimation Based on Poplar Seedling Geometry

**DOI:** 10.3390/s150612999

**Published:** 2015-06-04

**Authors:** Dionisio Andújar, César Fernández-Quintanilla, José Dorado

**Affiliations:** Institute of Agricultural Sciences, CSIC, Madrid 28006, Spain; E-Mails: cesar@csic.es (C.F.-Q.); jose.dorado@csic.es (J.D.)

**Keywords:** Kinect, angle of view, plant structure characterization, biomass assessment, energy crops

## Abstract

In energy crops for biomass production a proper plant structure is important to optimize wood yields. A precise crop characterization in early stages may contribute to the choice of proper cropping techniques. This study assesses the potential of the Microsoft Kinect for Windows v.1 sensor to determine the best viewing angle of the sensor to estimate the plant biomass based on poplar seedling geometry. Kinect Fusion algorithms were used to generate a 3D point cloud from the depth video stream. The sensor was mounted in different positions facing the tree in order to obtain depth (RGB-D) images from different angles. Individuals of two different ages, e.g., one month and one year old, were scanned. Four different viewing angles were compared: top view (0°), 45° downwards view, front view (90°) and ground upwards view (−45°). The ground-truth used to validate the sensor readings consisted of a destructive sampling in which the height, leaf area and biomass (dry weight basis) were measured in each individual plant. The depth image models agreed well with 45°, 90° and −45° measurements in one-year poplar trees. Good correlations (0.88 to 0.92) between dry biomass and the area measured with the Kinect were found. In addition, plant height was accurately estimated with a few centimeters error. The comparison between different viewing angles revealed that top views showed poorer results due to the fact the top leaves occluded the rest of the tree. However, the other views led to good results. Conversely, small poplars showed better correlations with actual parameters from the top view (0°). Therefore, although the Microsoft Kinect for Windows v.1 sensor provides good opportunities for biomass estimation, the viewing angle must be chosen taking into account the developmental stage of the crop and the desired parameters. The results of this study indicate that Kinect is a promising tool for a rapid canopy characterization, *i.e.*, for estimating crop biomass production, with several important advantages: low cost, low power needs and a high frame rate (frames per second) when dynamic measurements are required.

## 1. Introduction

Biomass has become one of the most frequently used renewable sources of energy in the last decade, with wood from trees being the most common biomass used to produce energy. Wood biomass can be derived from thinning and trimming as a regular sustainable woodland management practice or through short rotation coppice, *i.e.*, growing and harvesting fast growing tree species as energy crops. There are different species suitable for coppicing, being poplar (*Populus* spp.) one of the most commonly used species in Europe because it develops fewer and thicker stems than other species (e.g., willow) and therefore has a lower proportion of wood bark, thus it is considered as the most promising energy crop [1]. In addition, poplar is one of the most productive woody energy crops, with high yields and low demand for inputs [2]. Cost-efficient planning and management of these plantations would be greatly facilitated if traditional methods for estimating the volume, height and, ultimately, the biomass of poplar trees, which are based on measuring individual randomly selected trees and the subsequent application of equations for the estimation, were improved [3]. Consequently, improved technologies applied to poplar short rotation coppice systems are needed to optimize production costs and reduce the environmental impact. One of these technologies is automatic characterization of the plant canopy for estimating biomass and therefore choose the appropriate time to harvest. Furthermore, the description of morphology, physiology or some intrinsic characteristics leads to an improvement in plant structure knowledge and therefore the health and growth status of the plants. In recent years, the rapid improvement of plant phenotyping processes and the use of automated systems are providing valuable tools for comprehensive characterization of plant traits [4]. Many authors have emphasized the importance of accurate and efficient methods in order to obtain proper plant phenotyping [5,6]. The importance of a good model leads to a good understanding of the architecture of plants and how they intercept light for photosynthesis processes. The use of these models could affect different fields of research, from plant breeding [7,8], health status [8] or even yield or biomass estimation.

Numerous imaging and non-imaging sensors have been used to replace costly and time consuming traditional methods for plant characterization: Normalized Difference Vegetation Index (NDVI) sensors, fluorescence systems, Terrestrial Laser Scanners (TLS) sensors and RGB cameras. The simplest systems are based on NDVI sensors that can distinguish green plants from bare ground by a difference in light reflection in the bands of red and near infrared [9,10]; these sensors use a self-illuminated (active sensor) light source in the red and near infrared wavelengths, respectively. The NDVI sensors are mainly used for crop nutrient management [11], although they can also be used for stress determination. Even though these are not the best choice for biomass estimation, there is a relationship between high biomass values and NDVI saturation. RGB cameras have been widely used because their efficiency and high resolution. In addition, these cameras have the advantage over other sensors of having a simpler processing system and low cost. RGB cameras have been used in precision agriculture in phenology monitoring [12], vegetation structure description [13], biomass and nitrogen status determination [14], crop yield estimation [15] or weed crop discrimination [16]. Furthermore, fluorescence sensors have been mainly used to detect plant stress [17]. 3D models, particularly those ones obtained by TLS, have become popular in plant science in recent years [18]. There are several systems using 3D techniques such as radar systems [19,20], magnetic resonance or X-ray [21], ultrasonic systems [22,23], hemispherical photography [24], stereo-vision [25] and depth cameras [26]. Some advantages of depth cameras associated with their low cost and high frame rate (number of frames per second) are promoting their increased usage. These cameras can be divided into two groups: Time of Flight (ToF) and structured-light emission cameras. Structured-light scanners are the last revolution in 3D modeling, mainly due to the devices from two manufacturers who have launched two models on the market: the Microsoft Kinect and the Asus Xtion.

Various studies have been conducted to assess different types of 3D cameras for plant phenotyping. Paulus *et al.* [27] reconstructed the volumetric shape of sugar beet taproots and their leaves in order to compare the available cameras in the market. They showed their great potential for plant reconstruction, phenotyping and possible automated applications, suggesting that these cameras could replace some alternative high-cost tools, such as laser scanners. However, branch detection is not as accurate as with other methodologies such as the above mentioned radar or RGB images. Chéné *et al.* [28] also investigated the potential of these low cost cameras in plant phenotyping, concluding that Kinect sensors could successfully identify plant shape. These authors suggested the need for further research toward the acquisition of larger images able to include several plants. Very few studies have been conducted in woody plants to assess 3D cameras for biomass estimation. Correa *et al.* [29] and Jay *et al*. [30] used a Kinect sensor to estimate the foliar density of fruit trees in order to control a spraying system. Nock *et al.* [31] conducted a test using structured light emission cameras (Kinect and Asus Xtion) to test *Salix* branches at different distances, showing their possibilities and limitations for reconstruction of branching architecture of woody plants.

Although, a fully tree geometric characterization is nowadays possible, getting a proper reconstruction is sometimes unachievable or the reconstruction is time-expensive and cannot cover a big area. In this regard, most of previous research takes a small sample of the trees from a unique viewing angle, without considering that the position of the sensor is a key parameter since some tree dimensions (e.g., height, width) could be occluded because a wrong angle between plant and sensor is used. A plausible solution to plant characterization would be to use several sensors simultaneously, each oriented a viewing angle. However, poplar plants characterized by its high height (about 4 m in the second year of growth) and a narrow frame of plantation (3 m wide rows) would involve great difficulty to design a structure capable of supporting multi-sensors and avoiding vibrations under field conditions of the sensors located at the farthest position from the platform. Consequently, the first approach that we have proposed in this study was to assess the appropriate location (*i.e*., the relative angle between the sensor and tree) from which a Kinect sensor must be fixed to a stationary platform for better characterization of trees under field conditions. Additionally, this research analyzes the relationship between plant characteristics estimated from Kinect sensor and the actual characteristics of plants depending on the angle from which the sensor is positioned.

## 2. Materials and Methods

### 2.1. Sampling System

A low cost system for 3D modeling was tested. It combined a Kinect sensor and specific software for point cloud acquisition. The Kinect sensor was originally designed for games using Microsoft Xbox. However, multiple applications have been done using this sensor outside gaming environments. The sensor is a structured-light device integrated by an RGB camera, an infrared (IR) emitter and an IR depth sensor. The depth integrated sensor consists of the IR emitter combined with the IR camera, which is a CMOS sensor. The RGB camera is equipped with a 400–800 nm bandpass filter. The IR camera has a 850–1100 nm bandpass filter which captures the distorted light pattern used to compute a depth map based on triangulation. The system captures frames with a depth image resolution of 640 × 480 pixels by a structured light method with a rate of 30 frames per second. The accurate depth range is low when comparing with ToF systems. It has an accurate range from 800 mm to a maximum of 4000 mm. However, the Kinect for Windows v.1 can be switched to Near Mode which provides a range of 500 mm to 3000 mm. It also reaches object up to 8000 mm, but with very low accuracy. A different accuracy exists within the measurement range which is influenced by the distance between the object and the Kinect sensor, being this resolution lower close to the limits. In addition, the use of static objects leads to higher accuracy. The most important limitation for the use of structured light sensors in agriculture is that special conditions of illumination are needed for outdoor detection [25]. Low and high natural illumination does not allow collecting a sufficient number of points in the point cloud and color detection is not accurate enough. Since the sensor output creates a high number of frames per second and this information is in many cases redundant, the information could be used to automatically remove outliers in the point cloud of the 3D model.

The Skanect^®^3D scanning software was used for data acquisition. This software uses the Kinect Fusion algorithms to recreate a 3D point cloud from the depth video stream. Prior to the depth image acquisition some calibration tests were conducted. The Kinect sensor has an error in depth images which increases with a quadratic relationship between the sensor and the measured object distance [32]. This error varies from a few millimeters to several centimeters when the object is located at the maximum range. For the calibration test, a 2 m surveying rod was positioned close to the poplar tree as a reference. The rod was divided in red-or-white color sections of 10 cm. The proper distance for positioning the sensor was calculated by comparing the actual height (section of 10 cm) and the height measured at the rod section.

**Figure 1:**
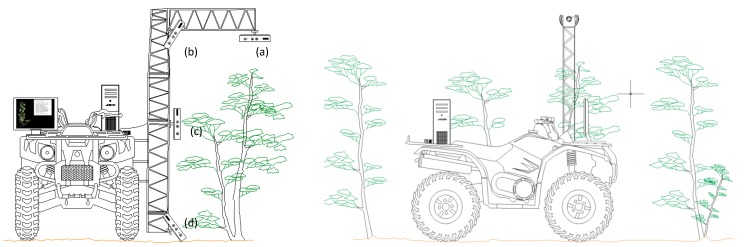
Schematic positioning of the sensor mounted on an ATV: (**a**) Top view (0°); (**b**) angular view (45°); (**c**) front view (90°); and (**d**) ground (−45°).

The assessment of appropriate angle between the sensor and poplar trees was conducted by placing the sensor in four different angular positions respect to the tree, thus obtaining depth images from fixed angles in a static mode. In this static mode, readings were acquired in individual trees through single instantaneous snap-shots, thus having a quick computational time. The Kinect sensor was mounted on an ATV with a 2 m structure and it was oriented to reach the proper angle (Figure 1). The angle of the sensor was tuned respect to the estimated tree mass center by positioning the four angles: top view (0°), 45° downwards view (45°), front view (90°) and ground upwards view (−45°). In addition, a study in dynamic mode was carried out by acquiring the meshes using a multi-angular mode-Kinect. In dynamic mode, the sensor was manually moved 360° around the tree while recording a point cloud. This multi-angular point cloud was then merged in a single cloud by software fusion. This cloud was used to compare the results with the static measurements.

### 2.2. Site Study and Plant Measurements

Three field and one lab measurements were conducted during 2014. The field was located in the experimental farm “La Poveda” (Arganda del Rey, Madrid, Central Spain, 618-m elevation). The location is characterized by a Mediterranean climate with cold winters and hot and dry summers. The crop was irrigated during spring and summer. Poplar trees were planted during spring 2013. The distance between poplar trees was 0.5 m and the row spacing was 3 m.

#### 2.2.1. One Year Old Plants

One year after planting, three field measurements were conducted at weekly intervals during July 2014, scanning 10 trees per sampling day. At sampling time poplar height ranged from 0.8 m to 2 m. Trees were randomly selected and, for the dynamic mode approach, the adjacent trees were cut and removed in order to facilitate the mobility of the sensor around the target tree.

**Figure 2:**
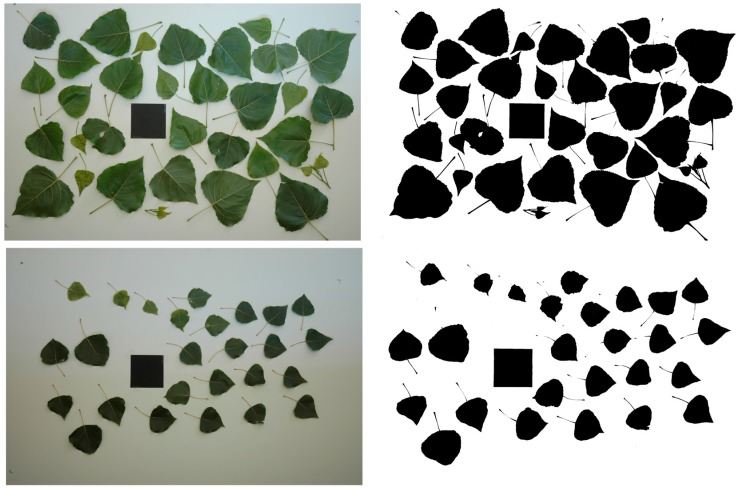
Leaf area images: RGB images and black and white transformation.

Immediately after the Kinect data were recorded, the actual height of trees was manually measured. Thereafter, trees were cut and divided in three sections: base, half height and top. These samples were processed in the lab for biomass determination (dry weight basis) and leaf area calculation. The comparison of these parameters with actual measurements was used to check the accuracy of the sensor to estimate the parameters of poplar trees. The leaf area was calculated from images consisting of all the leaves placed on a white surface. Images were acquired with a Nikon D70 (Nikon Corporation, Tokyo, Japan) camera fitted with a 50 mm Nikkor lens. It incorporated a 6.1 megapixel DX Format CCD image sensor that produces 3008 × 2000-pixel images. The RGB images were transformed to binary images. Excess green index (ExG = 2G-R-B) was applied, which gives a grey level image with green objects (plants) appearing bright, in contrast to objects with a different color which appear dark. Then, a threshold of 8% was applied to the ExG to separate the objects pixel-wise into foreground (plants) and background in a binary image. From this image, the values marked as black denoted leaf area and those pixels off the range, which corresponded to white, denoted the background. As a reference surface, a standard 100 cm^2^ square was also placed in the image in order to calculate, by correlation, the leaf area of each sample (Figure 2).

#### 2.2.2. One Month Old Plants

**Figure 3:**
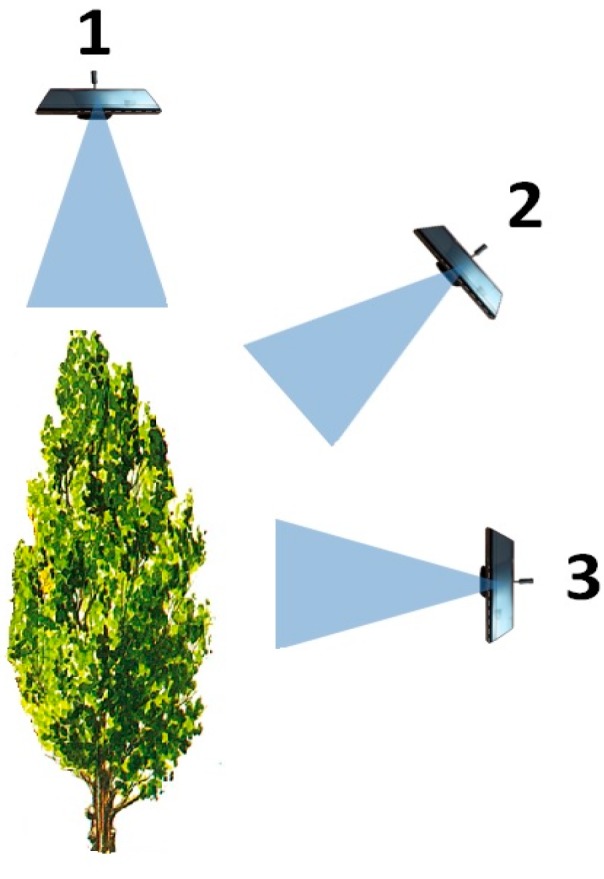
Schematic positioning of the sensor: (1) Top view (0°); (2) 45°; and (3) front view (90°).

A similar procedure to that explained above was used for the laboratory experiment in which a total of ten poplar plants was grown for a month under controlled conditions. The plant models were constructed with the Kinect sensor using three angles: top view (0°), 45° and front view (90°) (Figure 3).

The models were created in both, static (single shot) and dynamic (multi-angular) modes, in order to compare their results. Then, plants were processed for biomass determination (dry weight basis) and leaf area calculation following the previous methodology.

### 2.3. Data Processing

Meshes were processed off line in the open source software Meshlab^®^. The software includes tools which allow cleaning, managing and processing the obtained meshes. The meshes were processed in different steps: (1) Filtering and removal of outliers and noise reduction by filters. Individual points located 2 cm out of solid mesh were removed using statistical filters. Those outliers that were not removed by filters, were subsequently removed by a supervised visual procedure; (2) Cleaning the neighboring poplars. The target poplars, one year old, were isolated by removing some close plants inside the boxes occupied by the studied poplars, defined by its maximum height. Although some filters were tested to remove these parts, none proved useful because of the high similarity and proximity between the inter-row plants. Thus, a manual cleaning process was done by removing every leaf and branch which did not correspond with the studied plant; (3) Dividing plants into segments according to three sections: base, half height and top. The surveying rod located near the model allowed to divide the 3D model in three different sections according to the real divisions carried out in the poplar tree. The results of each division performed manually were three individual 3D models which were stored in order to compare with the actual values of biomass and leaf area for the corresponding section. The leaf and branch area of each division was calculated using Meshlab^®^ software. The model height was also measured and compared with the actual poplar height.

A similar procedure was done for smaller one month old poplars. Noise and outliers were removed with statistical filters and visual detection in Meshlab^®^ software. Since this test was carried out in lab conditions, neighbor plants were not present in any case. The 3D model was then used to measure the poplar height and this value was compared with the actual height. Similarly, the leaf and branch area was calculated with Meshlab^®^ software and compared with dry biomass and actual leaf area.

## 3. Results and Discussion

### 3.1. Relationship between Estimated and Actual Plant Parameters

One year old poplar trees differed in their architecture with some individual plants presenting two separated trunks. This case was considered as a unique tree and both tree trunks were included in the analysis. After the calibration test, the distance of one meter between sensor and tree was chosen for the final depth image acquisition. Since all poplars were planted at the same time and preliminary analysis did not show significant differences between sampling dates field measurements were grouped and analyzed all together.

In general, results for one year old trees showed that total plant biomass and tree height were always well estimated (correlations significant at 99%) by the sensor located in all positions, indicating the potential of depth cameras for plant phenotyping (Table 1). Since ground was visible in the mesh, height was properly measured with an error lower than 2 cm in all cases, except for those measurements taken from the top.

**Table 1:** Coefficients of correlation between actual measurements of biomass, area and height, and parameter estimation using Kinect sensor from different viewing angles in one year old poplar trees.

Viewing Angle	Leaf Biomass			Branch Biomass			Leaf Area			Total Biomass	Height
	Top	Half Height	Base	Top	Half Height	Base	Top	Half Height	Base	Full Tree	
0°	0.421	0.756 **	−0.065	−0.016	0.632 **	−0.017	0.201	0.062	−0.153	0.729 **	0.747 **
45°	0.662 **	0.896 **	0.579 **	0.249	0.748 **	0.549 **	0.746 **	0.720 **	0.829 **	0.799 **	0.979 **
90°	0.669 **	0.916 **	0.589 **	0.198	0.762 **	0.479 *	0.625 *	0.125	0.579	0.802 **	0.982 **
−45°	0.671 **	0.843 **	0.619 **	0.249	0.695 **	0.114	0.777 **	0.502	0.391	0.747 **	0.944 **
Multiangle	0.787 **	0.746 **	0.692 **	0.510 *	0.737 **	0.650 **	0.779 *	0.673	0.152	0.866 **	0.988 **

* Correlation significant at *p* < 0.05 level; ** Correlation significant at *p* < 0.01 level.

Regarding one month old plants, a significant correlation between estimated tree height by Kinect sensor and manually measured actual height was found (Table 2). Total biomass correlations were significant at 95% only in the case of top view. Viewing angles of 45° and 90° did not show good results in relation with most of the measured parameters. Only branch biomass was correlated for the front view (90°). In contrast with taller plants a year old, the top view (0°) showed the best results in tree characterization, with significant correlations in the cases of leaf area and total biomass (Table 2).

**Table 2:** Coefficients of correlation between actual measurements of biomass, area and height, and parameter estimation using Kinect sensor from different viewing angles in small poplars.

Viewing Angle	Leaf Biomass	Branch Biomass	Total Biomass	Leaf Area	Height
0°	0.738 *	0.051	0.663	0.891 **	0.923 **
45°	0.260	0.568	0.368	0.343	0.940 **
90°	0.010	0.849 **	0.215	0.011	0.944 **
Multiangle	0.759 *	0.724 *	0.845 *	0.853 **	0.924 **

* Correlation significant at *p* < 0.05 level; ** Correlation significant at *p* < 0.01 level.

### 3.2. Sensor Angle Comparison and 3D Analysis

**Figure 4:**
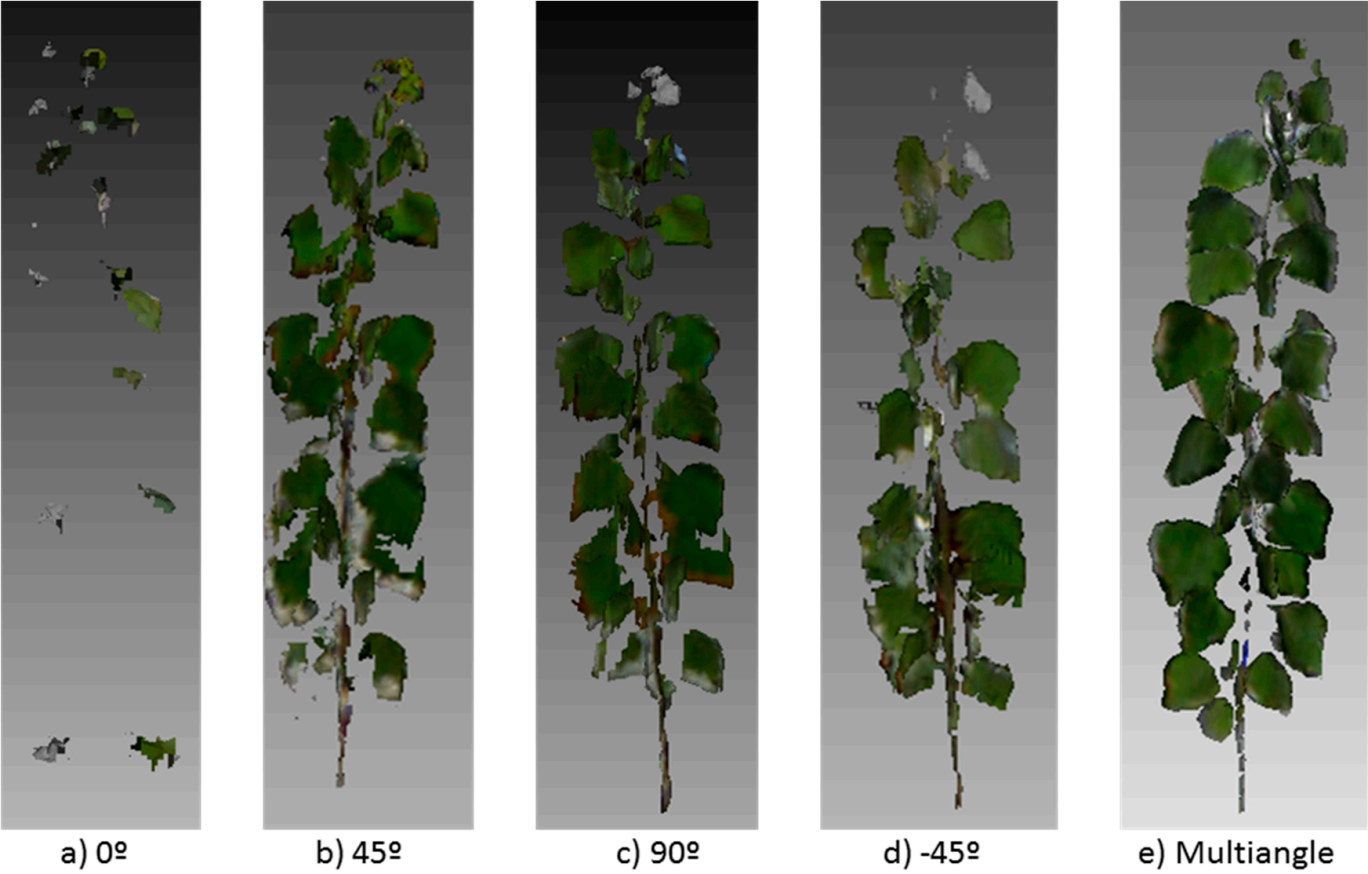
3D model obtained in one year old plants from a viewing angle: (**a**) 0°; (**b**) 45°; (**c**) 90°; (**d**) −45°; and (**e**) multiangle.

The two studied cases, one year old and one month old plants, led to different results concerning the best angle for the sensor. Indeed, the results indicated that sensor location is a key factor for a correct plant phenotyping. The top view (0°) showed poor results in the characterization of some parts of one year old trees. Although this view showed a significant correlation with leaf and branch biomass in the half height segment of the tree, it underestimated all the remaining parameters, probably due to the fact that top leaves occluded the rest of the tree (Table 1). The views from angles 45°, 90° and −45° showed good results in relation with most of the measured parameters. In the case of the 45° sensor location, only the top segment did not show a significant correlation with the Kinect measurement. This effect was probably due to the small branch diameter in the top segment (Figure 4). Nock *et al.* [31], trying to reconstruct the branching architecture of *Salix* plants with a similar sensor, found that the sensor accurately captured the diameter of branches >6 mm but small branches remained undetectable at certain distances. In the same way, the front view perfectly reconstructed most of the parameters of tree shape and biomass, except top branch biomass, probably due to the small branch diameter. Similar results were obtained for the −45° angle of view. 90° and −45°, did not show any significant correlation with leaf area. Finally, the multiangle approach, used as control, provided similar results to 45°, 90° and −45° angle of view, which indicates that a single shoot could be enough for tree characterization (Figure 4). Thus, the use of a top view is not justified in any case and the other three angles could be used depending on the target to study. Small ground robots could place the sensor at 45° to obtain depth models, while other vehicles such as ATVs or tractors, could locate the sensor at 45° or 90° which shows better results for branch estimations.

**Figure 5:**
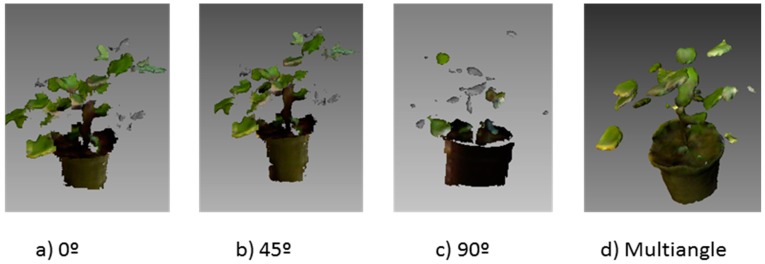
3D models obtained in one month old plants from a viewing angle: (**a**) 0°; (**b**) 45°; (**c**) 90°; and (**d**) multiangle.

According to these results, when considered plants of any size, not a fixed angle can be defined as the best location of the sensor in a vehicle. In the case of one year old poplars, the front angle represents one of the best locations. However, when plants are smaller (e.g., one month old plant. Figure 5), the front angle leads to poorer results. In contrast, the top view represents the best angle for small plants and the worst for the taller plants. A comparison of Figure 4c and Figure 5c shows the importance of choosing a proper angle for the right growing stage. Similar results were obtained in other crops using TLS sensors [33,34].

In cases with very low illumination or totally in the darkness, detection with a Kinect sensor can be improved by illuminating the crop with artificial light. Comparison of this sensor with a TLS sensor, although the latter is not influenced by ambient light, Kinect sensor has the advantage of obtaining a 3D cloud of points for each frame, while 2D TLS must be moved to obtain a 3D model. However, the precision of Kinect seems to be lower for the detection of thin branches (Figure 4 and Figure 5). In summary, when comparing TLS sensors and depth cameras, the latter have the advantage of their capability to record 3D color point clouds in a single shoot and, to detect plants structure in a faster and reliable way, with a very high spatial resolution. The new model that Microsoft is currently launching (Kinect for Windows v2) will improve the advantages of this sensor respect to Kinect v.1 and TLS. The new model includes a high-definition (HD) color camera and it uses the principle of ToF instead of structured light, which theoretically improving accuracy and penetration in plants for 3D characterization. This camera gives a cleaner depth image and smaller shadows compared to version 1 with the structured light approach. However, all these features are not important if the sensor is not correctly located. The location of the sensor is key to achieving the best results. Indeed, optimal performance depends on the sensor orientation and location, and it should be taken in consideration for plant detection, robot guidance or any other application. The measurements carried out in this study show the potential of this sensor to collect, to fuse and to analyze RGB-D three-dimensional properties of tree crops, with great application to several agricultural processes such as yield mapping and crop management, *i.e.*, irrigation, fertilization, and plant protection.

## 4. Conclusions

3D crop modeling using depth cameras is achievable. Single snap-shots showed the potential of structured light sensors to collect spatial and color information of surroundings to characterize the RGB-D three-dimensional properties of tree crops. The presented study showed that the best location depends on the growing stage. Tree structure, leaf density and leaf position respect to the sensor are important parameters that should be considered to allocate the sensor in a certain angle and position. This study concluded that small plants should be explored from top view, while the front angle represents one of the best locations for higher trees and the top angle leads to poorer results.

The Kinect sensor provides reliable data. The advantages of this type of sensor are the relatively low price and the ability to integrate it into real-time applications which could lead to an improvement in crop management. However, the possibilities of on-line operations need to be further explored using the best location and sensor angle in a vehicle. The vibrations in on-the-go operations could introduce noise in the readings and the development of algorithms to build the models and to extract useful information should be improved. Indeed, the sensor needs to be rugged to work outdoor in agricultural environments. In addition, the inoperability of this sensor under high illumination and the lower capacity to penetrate inside the measured plants should be explored.
